# Anticoagulants, Their Laboratory Monitoring, and Reversal

**DOI:** 10.3390/hematolrep18050067

**Published:** 2026-09-20

**Authors:** Mohammad Barouqa, Marianne E. Yassa, Nestor Dela Cruz, Maha Babker

**Affiliations:** Department of Pathology, University of South Alabama, 2451 University Hospital Dr., Mobile, AL 36617, USA; meyassa@health.southalabama.edu (M.E.Y.); mbabker@health.southalabama.edu (M.B.)

**Keywords:** anticoagulants, anticoagulation testing, anticoagulant reversal

## Abstract

Anticoagulant therapy has undergone substantial evolution over the past century, progressing from early parenteral agents such as heparin to vitamin K antagonists (VKAs) and, more recently, to direct oral anticoagulants (DOACs). This review provides an overview of the major classes of anticoagulants, including unfractionated heparin (UFH), low molecular weight heparin (LMWH), fondaparinux, direct thrombin inhibitors, VKAs, and DOACs, with emphasis on their mechanisms of action, clinical applications, laboratory monitoring, and reversal strategies. While UFH remains widely used in acute settings, its unpredictable pharmacokinetics and risk of heparin induced thrombocytopenia have led to increased use of LMWH and fondaparinux. VKAs, historically the cornerstone of oral anticoagulation, are limited by variable dosing requirements and the need for routine monitoring. In contrast, DOACs have transformed anticoagulation management through predictable pharmacokinetics, fixed dosing, and improved safety profiles, and are now first line therapies for several thromboembolic conditions. The development of targeted reversal agents, including protamine, vitamin K, prothrombin complex concentrates, idarucizumab, and andexanet alfa, has significantly enhanced the safety of anticoagulant therapy in the setting of major bleeding or urgent procedures. Laboratory assessment remains critical for selecting anticoagulants and evaluating clinical scenarios, with evolving roles for anti-Xa assays, thrombin-based assays, and mass spectrometry techniques. Emerging anticoagulants targeting factors XI, XII, and XIII represent a promising future direction with the potential to further reduce bleeding risk while maintaining efficacy. Overall, continued advances in anticoagulant pharmacology, monitoring, and reversal strategies are driving a shift toward safer and more individualized patient care.

## 1. Introduction

Anticoagulants have evolved over more than a century, from hirudin in leech saliva discovered in the 1800s to discoveries like heparin and warfarin to precisely designed direct oral anticoagulants (DOACs). In parallel, clinicians have developed increasingly targeted reversal agents to manage life threatening bleeding and urgent surgery.

### 1.1. Heparin, Low Molecular Weight Hepain and Heparinoids

The early anticoagulants were parenteral agents and included Heparin (UFH), a naturally occurring glycosaminoglycan. Early observations of liver associated anticoagulant activity were reported by Doyon and colleagues in 1911, who described an anticoagulant that they termed “antithrombine” [1]. In 1916, McLean, working in Howell’s laboratory, independently identified anticoagulant activity in extracts from canine liver [2]. Howell and Holt subsequently expanded upon this work and introduced the term “heparin” in 1918. Following further studies and development, heparin entered clinical use in the 1930s and became a standard injectable anticoagulant, although its use required frequent laboratory monitoring and dose adjustment [3]. Heparin acts as a catalyst for the antithrombin III (ATIII) which inhibits thrombin and other coagulation factors like factors X, IX, XI and XII [2], and later its administration routes were developed to include subcutaneous route that allowed its use for prophylactic measures, while Intravenous use was reserved for acute settings [3]. Because heparin has unpredictable pharmacokinetics and requires ongoing therapeutic monitoring, there was a need to develop a more reliable alternative. This led to the creation of low molecular weight heparin (LMWH), produced through chemical and enzymatic depolymerization [4]. LMWH reduced the need for monitoring and frequent laboratory testing. However, LMWH is excreted by kidneys, which limits its use in patients with renal impairment. Furthermore, it is partially inhibited by protamine, UFH antidote [5]. Heparin therapy is associated with a spectrum of adverse events, ranging from minor bruising to life threatening hemorrhage. Among its most significant complications is heparin-induced thrombocytopenia (HIT), which occurs in up to 5% of patients. HIT is characterized by the formation of IgG, IgA, and IgM antibodies against the heparin–platelet factor 4 (PF4) complex. Of these, IgG antibodies are primarily responsible for platelet activation upon binding to the complex, leading to a paradoxical prothrombotic state. HIT is more commonly observed with unfractionated heparin (UFH) than with low molecular weight heparin (LMWH) [3,6]. Low molecular weight heparin (LMWH) and fondaparinux have largely replaced unfractionated heparin (UFH) over time for many indications due to their more predictable pharmacokinetics, ease of subcutaneous administration, and improved safety profiles, particularly with respect to a lower risk of heparin-induced thrombocytopenia (HIT) [7,8].

Danaparoid is an injectable heparinoid anticoagulant used for the last 40 years, mainly to prevent and treat thromboembolism in patients where heparin is contraindicated especially in HIT [9]. It is constituted of a mixture of mostly heparan sulfate with small amounts of dermatan and chondroitin sulphates. Its mechanism of action is to inhibit factor Xa mainly and more than thrombin [10]. Clinically, it has been used for prophylaxis and treatment of deep vein thrombosis, for HIT with or without thrombosis, in high risk settings such as cardiac surgery, pregnancy, renal failure, and vaccine induced immune thrombosis, and to maintain patency of extracorporeal circuits [9]. Since 2002, Danaparoid is not available in the U.S. due to shortage of the drug substance required for its manufacture, but it is still used in several other countries across the world [11].

### 1.2. Vitamin K Antagonists

In the 1920, the ingestion of spoiled sweet clover hay lead into hemorrhagic disease in livestock in North America which lead in 1939 to the discovery of dicumarol, a coumarin derivative, as the causing agent without fully understanding the underlying mechanism at University of Wisconsin in Madison [2]. These findings lead into discovering vitamin K antagonists (VKAs) which dominated the oral anticoagulant therapies for more than 50 years [4]. VKAs exert their anticoagulation effect by inhibiting the hepatic gamma carboxylation of vitamin K dependent coagulation factors (factor II, factor VII, factor IX and factor X) by competitively binding to the vitamin K epoxide reductase complex 1 (VKROX1) resulting in the formation of proteins induced by vitamin K absence (PIVKA) which limit the coagulation cascade [5]. Warfarin, a derivative of dicumarol, has become a cornerstone in the treatment of thromboembolic events and named after the Wisconsin Alumni Research Foundation [6]. Patients receiving this treatment still need regular checks for the International Normalized Ratio (INR) especially that it is affected by age, weight, illnesses and other interacting medications [7]. The use of warfarin carries a spectrum of complications ranging from common bleeding to rare but severe cutaneous adverse reactions [8]. Among the most severe of these is warfarin induced skin necrosis with an estimated incidence of 0.01% to 0.1% and typically manifests with the first 3 to 10 days of therapy initiation [9]. It is mainly caused by the paradoxical transient to hypercoagulable state caused by the depletion of proteins C and S, which have shorter half-lives compared to the vitamin K dependent procoagulant factors [10].

### 1.3. Parentral Direct Thrombin Inhibitors and Indirect Factor Xa Inhibitors

Bivalirudin, argatroban and fondaparinux are relatively new anticoagulants that were developed to inhibit specific factors in the coagulation cascade. They are mainly indicated when heparins are contraindicated for instance in HIT. Fondaparinux is a synthetic pentasccharide that binds antithrombin and selectively inhibit factor Xa but not thrombin [11,12]. Argatroban and bivalirudin are direct thrombin inhibitors (DTIs) that inhibit both soluble and clot-bound thrombin and do not require antithrombin with bivalirudin is cleared both enzymatically and renally and argatroban is hepatically cleared [13]. DTIs have also emerged as alternative anticoagulants for patients receiving extracorporeal membrane oxygenation (ECMO), particularly when heparin cannot be used because of HIT or acquired antithrombin deficiency. Argatroban has been increasingly utilized in this setting, with several studies demonstrating bleeding and thromboembolic complication rates generally comparable to those observed with unfractionated heparin. However, considerable variability in dosing and therapeutic monitoring remains which necessitates the need for larger prospective studies to establish optimal anticoagulation strategies [14].

### 1.4. Direct Oral Anticoagulants (DOACs)

Since their introduction around 2010, direct oral anticoagulants (DOACs), including dabigatran, rivaroxaban, apixaban, edoxaban, and betrixaban, have transformed the management of thrombotic disorders. Their predictable pharmacokinetics and oral route of administration improve ease of use and patient compliance. Consequently, they are now considered first line therapy for multiple indications, including atrial fibrillation, venous thromboembolism, and thromboprophylaxis in orthopedic surgery [15,16]. DOACs have different mechanisms of action which include inhibiting the catalytic site factor Xa for rivaroxaban, apixaban, edoxaban and betrixaban whereas dabigatran directly inhibits thrombin by binding its catalytic site, blocking the conversion of fibrinogen to fibrin and thereby preventing clot formation. Dabigatran, the first agent introduced in this class, along with other DOACs, demonstrates an improved safety profile compared with VKAs and heparins, owing to its fixed dosing regimen and the absence of a requirement for routine coagulation monitoring [16,17]. Despite these advantages, the safety profile of DOACs remains closely linked to their anticoagulant effect, particularly when therapy is extended beyond conventional treatment durations. Studies showed that extended duration of thrombophylaxis with agents such rivaroxaban and apixaban, as well as enoxaparin, demonstrated benefits; however, these benefits were accompanied by increased bleeding, raising concerns about their use for extended anticoagulation [18,19].

### 1.5. Emerging Anticoagulants

Currently, there is a new generation of anticoagulants being evaluated by clinical trials and include factor XI, factor XII and factor XIII inhibition. These molecules are mainly monoclonal antibodies, oligonucleotides, peptides, aptamers and natural inhibitors [20,21]. Overall, these agents are moving from concept to real clinical options but are not yet a full replacement for existing anticoagulants. Among these targets, inhibition of FXI or activated FXI (FXIa) has advanced in clinical development. FXI contributes to amplification and propagation of thrombus formation but appears to have a comparatively limited role in normal hemostasis, as individuals with congenital FXI deficiency generally have a milder and more variable bleeding phenotype than patients with deficiencies of factors VIII or IX. This observation has supported the concept that FXI/FXIa inhibition may provide antithrombotic efficacy with less bleeding than conventional anticoagulants. Several studies have evaluated FXI/FXIa inhibitors in settings including venous thromboembolism prevention after orthopedic surgery, atrial fibrillation, end-stage kidney disease, and secondary prevention following cardiovascular or cerebrovascular events, although efficacy has varied according to the agent and clinical indication. FXII inhibition represents another potentially attractive strategy because FXII is important for contact pathway-mediated thrombosis while congenital FXII deficiency is not associated with a clinically significant bleeding tendency. However, its clinical development remains less advanced than that of FXI inhibitors. [22,23,24,25].

## 2. Laboratory Monitoring and Anticoagulant Reversal

### 2.1. Heparin Reversal and Laboratory Monitoring

Protamine is the only approved reversal agent for UFH, and it is also used for LMWH due to its partial inhibition capacity. They are histones, rich in arginine polypeptides and have an integral part in stabilizing DNA. Their molecular weight ranges from 4000 to 5000 Da and contain 50 to 110 amino acids making their molecular weight low [26]. Arginine is an integral amino acid in their structure and constitutes around 70% of their structure. Such configuration allows them to bind to negatively charged molecules like heparin. Pharmaceutical protamine is derived from fish (mainly from salmon sperm), and it is used medically in sulphate or chloride forms intravenously to electrostatically bind to UFH rapidly. Given its pharmacokinetics, it has a short half life which is estimated to be approximately 5 min [27]. LMWH has a longer half life compared to UFH, but smaller fragment size and different sulfonation, and with protamine depending on the molecular size for neutralization, it is justifiable that UFH is neutralized with protamine while LMWH is not [28].

Several adverse events have been associated with protamine administration and included hypotension, pulmonary hypertension, bronchospasm and shock [29]. However, the exact mechanisms associated with its administration and whether it is an immunologic response vs. direct effects on the cardiovascular system is still unclear [27].

In the clinical laboratories, activated partial thromboplastin time (aPTT) has been historically used to monitor UFH since 1970. However, aPTT exhibits several limitations that render its use in several clinical settings, and they are related mainly to pre-analytical and analytical factors which are mainly related to reagents, instruments and interference from lupus anticoagulants or elevated factor VIII levels [30,31]. Given these limitations, the anti-Xa assay emerged as a more specific test compared to aPTT as it can directly measure heparin’s functional activity by analyzing its ability to inhibit exogenous factor Xa [31]. Anti-Xa assay provides a more stable dose response curve compared to aPTT, and patients require fewer dosage adjustments as the therapeutic range for anti-Xa is reached faster (0.3–0.7 U/mL) [32]. During procedures like cardiopulmonary bypass or percutaneous coronary intervention. Activated clotting time (ACT) remains the preferred methodology of testing for high dose heparin [32,33]. However, ACT is affected by several factors that include hemodilution, hypothermia and platelet dysfunction which can lead into poor correlation with actual plasma heparin concentrations. These tests are capable of to maintain its concentration within the therapeutic window [34]. For HIT, laboratory testing include PF4 enzyme linked immunosorbent assay (ELISA), heparin induced platelet aggregation (HIPA) and serotonin release assay (SRA), mainly used for confirmation [35].

### 2.2. VKA Reversal and Laboratory Monitoring

VKAs, most notable warfarin, has been the focus of clinical research for the last decades, and because their duration of action are dictated by the half-lives of the vitamin K dependent factors inhibited [36]. Vitamin K has been the specific antagonist for VKAs as it accelerates the hepatic biosynthesis of new clotting factors, and it is usually administrated either intravenously with an onset of action of 1 to 2 h with a full effect after 12 to 24 h or orally what is usually reserved for asymptomatic supratherapeutic INRs with an onset of action that spans from 6 to 12 h [37]. IV vitamin K is mainly associated with very rare but severe hypersensitivity reaction and a boxed warning exists when it is administrated [38]. Furthermore, the slow onset and length of time required to reach maximal effect render vitamin K usage unsuitable to reverse warfarin in critical cases such as major trauma or massive bleeding. Fresh Frozen Plasma (FFP) has been historically used for rapid reversal, as it provides the coagulation factors inhibited by VKAs. However, the use of FFP in reversing INR is associated with dose dependent risk where pulmonary complications in 20% of patients were reported especially when more than 3 units of FFP are used [39]. In addition, the use of FFP can be associated with logistical issues related to finding required number of ABO matched units and delays in thawing the units [40]. Prothrombin Complex Concentrates (PCCs) are now considered the gold standard for emergent VKA reversal. Those reagents are plasma derived, virus inactivated concentrated containing concentrated amounts of vitamin K dependent factors [41,42]. The main concentrates used is the 4-Factor PCC (4FPCC) which contains factors II, VII, IX and X along with proteins C and S which is mainly used for rapid reversal of VKA associated major bleedings and showed an efficacy of achieving a target INR of ≤1.5 in 73% to 80% of patients making them superior to the 3-Factor PCC which contains lower levels of factor VII [42,43]. Protein C and protein S play a vital role in balancing the thromboembolic complications of the reversal reagents [44].

Prothrombin time remains the base for the primary assay for monitoring these reagents. However, because PT reagents, specifically the thromboplasitns, vary in their sensitivity to VKA induced factor deficiencies, the international normalized ratio (INR) was introduced to standardize results among different laboratories [45]. With applying INR, the therapeutic target can be typically followed up in the laboratory every 4 weeks and dose adjusted as needed to avoid the risks of over or under coagulating patients, and recent publications suggest that the frequency of testing can be extended up to 12 weeks in patients with stable INR control [46,47]. Point of care (POC) testing for INR monitoring has been an evolving field as it allows patients self testing and reduce turn around time in clinical laboratories. However, laboratory based verification is still recommended for critical decisions due to the reported discordance between POC and traditional plasma based assays especially when the INR value is higher 2.0–3.0 [48].

### 2.3. DTI and Fondaparinux Reversal and Laboratory Monitoring

A primary clinical limitation of both bivalirudin and argatroban is the lack of a specific FDA approved pharmacologic antidote or reversal agent available [49,50]. However, due to their short half lives, the first step in managing bleeding is to immediately stop their infusion, with special consideration to the renal and hepatic functions of patients before their use. Similar to DTIs, fondaparinux has no specific reversal reagent, and its reversal is focused on supportive care and in extreme cases, the use of high dose 4 factor PCC or recombinant factor VIIa, which may partially restore thrombin despite persistent anti-Xa activity [51].

In the clinical laboratory, argatroban and bivalirudin are typically monitored either by aPTT or ACT (mainly during procedures) for dose optimization as both can falsely elevate PT/INR and interfere with clot based assays [52]. For quantification purposes, specialized assays which include dilute thrombin time, ecarin clotting time and chromogenic anti-IIa can be used and generally require a specialized coagulation laboratory to perform [13,34]. Fondaparinux is usually followed by specialized anti-factor Xa chromogenic assay (specially calibrated for fondaparinux standards) and performed for specific clinical indications like in pediatric patients, renal impairment, pregnancy, obese or underweight patients or having an unexpected hemorrhage or thrombosis event [53].

### 2.4. DOAC Reversal and Laboratory Monitoring

The reversal of DOACs includes the use of specific antidotes and non specific agents when antidotes are not available. For dabigatran, its reversal agent is Idarucizmab, which is a humanized monoclonal antibody fragment with high affinity to dabigatran and provides immediate and complete anticoagulant reversal effect [54]. On the other hand, Andexanet Alfa which is a recombinant and inactive form of factor Xa is the main antidote for other factor Xa inhibitors and acts as a competitive inhibitor or decoy for them to bind to. It has a rapid onset between 2 to 5 min with a half life span between 30 to 60 min. The serine to alanine mutation in its active site plays an integral role in eliminating its capacity to cleave prothrombin to thrombin, but reserves its capacity bind Factor Xa inhibitors [55]. Although neutralizing antibodies to Andexanet have not been observed, low levels of non-neutralizing antibodies can still develop and clear within 15 to 30 days after administration indicating that andexanet has minimal immunogenic capacity [56,57]. One of the major challenges of clinically adopting Andexanet and Idarucizmab in healthcare systems is their high-cost limiting their rapid availability. Routine coagulation monitoring for DOACs is not necessary because of their predictable pharmacokinetics and broad therapeutic window [58]. However, they are usually followed up in specific clinical conditions which include emergencies like major bleeding, urgent surgery, acute ischemic stroke before thrombolysis, extremes of weight, renal/hepatic dysfunction, treatment failure and suspected overdose [59,60,61]. Liquid chromatography-mass spectrometry (LC-MS/MS) is considered the gold standard method for evaluating DOAC in clinical laboratories. Nevertheless, it has several limitations that include delays and expensive instrumentation [58]. PT, aPTT and TT are not reliable for quantification due to variable sensitivity by drug and reagent. For dabigatran, dilute thrombin time or ecarin based assays are used to exclude clinically relevant levels and show linear correlation with the anticoagulant plasma concentrations across a wide range [58,62]. Other factor Xa inhibitors are followed up in the laboratory using calibrated chromogenic anti-Xa assays with drug specific calibrators [63]. Prothrombin time (PT) may be prolonged by rivaroxaban and edoxaban, but its sensitivity is reagent dependent and often too low to exclude therapeutic levels especially for apixaban which frequently leaves the PT within normal limits [64]. Regular renal function testing is essential when using DOACs, as all are eliminated through the kidneys to varying extents. Renal clearance accounts for approximately 80% of total elimination for dabigatran, 50% for edoxaban, 35% for rivaroxaban, and 27% for apixaban [65,66,67].

The anticoagulant class, their mechanism of action, clinical laboratory testing and reversal reagents are summarized in Table 1.

## 3. Conclusions

Anticoagulant therapy has evolved from early parenteral agents such as heparin and vitamin K antagonists to modern direct oral anticoagulants (DOACs) that offer predictable pharmacokinetics, fixed dosing, and improved safety profiles. While traditional agents remain important, their limitations, such as the need for frequent monitoring and variable responses, have driven the development of newer therapies and more precise laboratory assays. Advances in reversal strategies, including specific antidotes and optimized use of prothrombin complex concentrates, have further improved the management of bleeding complications. Challenges persist in certain clinical scenarios and patient populations. Emerging anticoagulants targeting factors XI, XII, and XIII hold promise for reducing bleeding risk while maintaining efficacy, highlighting a continued shift toward safer, more individualized anticoagulation management.

## Figures and Tables

**Table 1:** Summary of Anticoagulant Class, Mechanism of Action, Monitoring and Reversal Agent/s.

Anticoagulant Class	Mechanism of Action	Monitoring	Reversal Agent/s
Unfractionated Heparin (UFH)	Enhances antithrombin → inhibits thrombin (IIa), Xa, IXa, XIa, XIIa	aPTT, anti-Xa, ACT (during procedures)	Protamine (complete reversal)
Low Molecular Weight Heparin (LMWH)	Enhances antithrombin → primarily inhibits Xa	Usually not routinely performed (anti-Xa in special cases)	Protamine (partial)
Synthetic Pentasaccharide	Selective indirect Xa inhibition via antithrombin	Anti-Xa (special cases)	None (PCC off-label)
Direct Thrombin Inhibitors (DTIs)	Direct inhibition of thrombin (free + clot-bound)	aPTT, ACT	None
Vitamin K Antagonists (VKAs)	Inhibits VKORC1 → reduce/inhibit factors II, VII, IX, X	PT/INR	Vitamin K, PCC, FFP
Direct Oral Anticoagulants (DOACs)-Dabigatran	Direct thrombin inhibition	None routinely (dTT, ECT if needed)	Idarucizumab
DOACs-Factor Xa inhibitors	Direct Xa inhibition	Anti-Xa (drug-specific)	Andexanet alfa, PCC

## Data Availability

No new data were created or analyzed in this study. Data sharing is not applicable to this article.
